# Composite reaction time and variability correlate with whole-brain white-matter characteristics

**DOI:** 10.1162/IMAG.a.1303

**Published:** 2026-07-17

**Authors:** Eirini Messaritaki, Craig Hedge, Pedro Luque Laguna, Carolyn B. McNabb, Derek K. Jones, Petroc Sumner

**Affiliations:** Cardiff University Brain Research Imaging Centre, School of Psychology, Cardiff University, Cardiff, United Kingdom; School of Psychology, Aston University, Birmingham, United Kingdom

**Keywords:** processing speed, intelligence, cognitive efficiency, connectivity, graph theory, information

## Abstract

The relationship between processing speed and brain network characteristics has been widely studied, yet the results remain inconsistent. While many studies have linked processing speed to the microstructure of white matter, discrepancies arise due to differences in the tasks used, behavioral measures assessed (based on raw reaction time or modeled processing speed), and specific white-matter tracts considered. To address these challenges and clarify any relationship between individual differences in speed and white-matter brain networks, we present a pre-registered analysis using a large (N = 159) dataset, incorporating state-of-the-art MRI data acquired from a high-gradient 3T Connectom scanner. We combine data from three reaction-time tasks to create composite measures of cognitive performance, mitigating the limitations of experiment-specific analyses. Alongside classic behavioral measures of mean reaction time, reaction-time variability, and accuracy, we applied the drift–diffusion model to derive the common metric of modeled processing speed, drift rate, as well as accompanying parameters of boundary separation, and non-decision time. Using general linear models, we explored the relationship between these parameters and the whole-brain and task-specific structural networks of the brain, weighted by volume-normalized streamline counts and myelin water fraction. Our results revealed negative associations between the global efficiency of streamline-weighted networks and both mean reaction time and reaction-time variability (β = -0.18/-0.21, *p* = 0.025/0.01 for whole-brain and β = -0.18/-0.18, *p* = 0.028/0.022 for the task-specific network). Effect sizes were small, consistent with other pre-registered assessments of brain–behavior correlations. These effects were not captured by decision model parameters signaling a note of caution for the assumed interpretation of these parameters. The significant association with reaction-time variability was robust to controlling for age, while age captured significant variance in the association with mean reaction time. This may imply that physiological changes associated with age would be an avenue for research to uncover mechanisms relating structure to reaction time. In sum, we attempted a state-of-the art clarification of whether structural brain organization is associated with speed in common cognitive tasks, and we found a small association with reaction-time variability and mean reaction time (and age).

## 1 Introduction

Individual differences in cognitive performance across tasks can be partially explained by a single underlying factor, general intelligence (“g”) (Deary, 2012; Jensen, 1998; Spearman, 1904). “g” correlates with processing speed, measured through reaction times, inspection times, and memory retrieval (Jensen, 2006; Sheppard & Vernon, 2008). This has led to the hypothesis that processing speed is a fundamental neuro-cognitive property underlying higher-order cognitive abilities (Jensen, 1993; Schubert et al., 2017). One proposed mechanism is that processing speed reflects the conduction velocity of action potentials in white matter (Bartzokis et al., 2010). Consistent with this, theories of intelligence suggest that the microstructural properties of white-matter tracts linking parietal and frontal brain areas enable efficient cognitive operations (Jung & Haier, 2007). Previous studies examining processing speed and white matter have largely relied on simple behavioral measures (e.g., mean reaction time) and examined isolated tracts, failing to capture the complexity of cognitive performance and brain microstructure. Here, we improve upon these approaches by integrating cognitive modeling, which attempts to dissociate distinct components of reaction time (RT), with graph theoretical analysis of networks using advanced microstructural imaging techniques.

Cognitive processes rely on transport of signals along the white-matter tracts that connect brain areas (Bullmore & Sporns, 2009; Lynn & Bassett, 2019). Signal transmission is affected by factors such as synaptic connections (Fornito et al., 2015) and myelination (Drakesmith et al., 2019; Tolhurst & Lewis, 1992). Accordingly, white-matter microstructural characteristics should correlate with processing speed and efficiency. Existing studies have reported correlations between fractional anisotropy (FA) of the diffusion tensor and both mean RT and RT variability (Booth et al., 2019; Haász et al., 2013; Kievit et al., 2016; Kuznetsova et al., 2016; McCormick et al., 2023; Penke et al., 2010, 2012), though the FA lacks a direct mapping to biological factors such as myelination and axonal density.

Neurophysiological evidence supports the role of white matter in cognitive speed, though not necessarily at a whole-brain level. Event-related potentials (ERPs) show that early sensory components (N1, P1) are weakly associated with g, whereas later processing stages (P2, N2, P3) show stronger associations (Schubert et al., 2017). It was, therefore, argued that correlation between processing speed and g specifically reflects the speed of communication between regions responsible for higher-order cognitive processes.

Here, we aim to provide a more definitive analysis of the association between white-matter network characteristics and cognitive performance using a larger cohort and more advanced microstructural imaging. The use of high-gradient diffusion MRI enhances the fidelity of both microstructural metrics and tractography reconstructions, giving us the best possible chance of detecting associations between behavioral measures and network characteristics. We treat the white matter as a network (Bullmore & Sporns, 2009; Rubinov & Sporns, 2010; Sporns, 2010), using graph theoretical metrics to estimate network efficiency (Avena‑Koenigsberger et al., 2017; Seguin et al., 2020), moving beyond traditional studies focused on individual tracts or whole-brain FA (Karahan et al., 2019; Kuznetsova et al., 2016; Madden et al., 2009; Tuch et al., 2005; Turken et al., 2008). Clinical studies suggest that slower processing speed in aging (Wen et al., 2011), brain injury (Caeyenberghs et al., 2014), and diabetes (Reijmer et al., 2013) is associated with such metrics (specifically higher characteristic path length and/or lower global efficiency), indicating that there may be similar patterns in healthy adults. A recent study on healthy participants (Imms et al., 2021) showed that processing speed in a local–global task was correlated with shorter path length in a task-specific subnetwork. Therefore, we apply the approach both to whole-brain and task-specific networks.

On the behavioral side, there is currently no consensus on what the optimal measure of processing speed is. Some studies have used a single task (Boekel et al., 2015; Forstmann et al., 2010, 2011; Imms et al., 2021; Karahan et al., 2019; Madden et al., 2009), while others have used multiple tasks and created composite measures (Haász et al., 2013; Kuznetsova et al., 2016; Penke et al., 2010, 2012). Following this, we administered three different choice–reaction-time tasks to capture processing speed across a range of contexts. Next is the question of the most appropriate measure to extract from the tasks. Mean RT is most common, but Eysenck (1982) argued that variability in RT should be considered the basic property of more intelligent brains. In his view, slower mean RTs were simply a consequence of more variable reaction-time distributions with a longer tail. Empirically, mean RT and intraindividual variability in RT are highly correlated (r = .6) but each accounts for some unique variance in measures of g (Jensen, 1992). However, their relative functional significance remains unclear, and neither is a pure measure of processing speed: they are influenced by speed–accuracy trade-offs, perceptual-motor speed, and reflect multiple cognitive components (Miller & Ulrich, 2013; Ratcliff & Rouder, 1998; Wickelgren, 1977).

Researchers have attempted to address these issues using models of simple decision making, such as the drift–diffusion model (Ratcliff, 1978), to extract parameters thought to more closely reflect the efficiency of information processing (drift rate), the extent to which participants strategically favor speed or accuracy (boundary separation or response caution), and the duration of non-decisional sensory and motor processing. If we accept the assumptions of these models at face value, then the drift rate parameter is expected to capture task-relevant processing speed. Indeed, some behavioral studies have observed higher correlations between g and drift rates than between g and raw measures of RT (for an overview, see Frischkorn et al. (2019)). However, boundary separation and non-decision time may also be of interest, given reports that these two parameters largely account for age-related slowing (Ratcliff et al., 2010; Theisen et al., 2021), and that boundary separation differences may better account for connectivity differences previously attributed to processing speed, such as between striatum and pre-supplementary motor area (Forstmann et al., 2011).

Finally, it is important to consider the role of aging, both as an expected correlate of reaction time and its variability, and as a potential clue to the types of physiological and cognitive differences that may be important in any measured correlation between white-matter networks and cognitive task performance. Chronological age is a proxy for a multitude of brain-related variables that change over time (Hedden et al., 2016; Salthouse et al., 2015), of which white-matter network characteristics is one (Madden et al., 2020; Merenstein et al., 2023). One testable possibility is that the well-known correlation between age and RT could be mediated by network characteristics measured here, following previously reported mediation by FA and structural or functional network metrics for age’s correlations with processing speed, executive function, and fluid cognition (Hedden et al., 2016; Madden et al., 2020; Merenstein et al., 2023). Therefore, we treat age as a clue to help unpack any association between white-matter networks and reaction time.

In summary, by integrating advanced white-matter measurement and network analysis, multi-task behavioral assessment, and cognitive modeling, this study aims to refine our understanding of whether and how processing speed is reflected in network characteristics at a biological level. In overview, participants performed three reaction-time tasks and we created cross-task measures of mean RT, RT variability, and accuracy. We also used the drift–diffusion model to derive parameters of processing speed (drift rate, our main interest)), as well as accompanying parameters of boundary separation, and non-decision time. Participants were scanned using a high-gradient 3T Connectom scanner and we extracted whole-brain and task-specific structural networks, weighted by volume-normalized streamline counts and myelin water fraction. In the final step, we used general linear models to elucidate any relationship between the network metrics and the cognitive performance measures/parameters.

## 2 Methods

### 2.1 Data collection

Multi-modal data were collected for the Welsh Advanced Neuroimaging Database (WAND) study (McNabb et al., 2024, 2025) at the Cardiff University Brain Research Imaging Centre (CUBRIC). The data collected for the modalities used in this work are described below. The analysis methods were described in the pre-registration report deposited in the Open Science Framework (Messaritaki et al., 2023) and the additional document describing a correction to the methodology (https://osf.io/kzw4g, more details provided below).

#### 2.1.1 Participants

The study recruited 170 healthy participants between 18 and 63 years of age. Exclusion criteria for taking part in the study were (McNabb et al., 2025) diagnosis of any heart or breathing problem; high blood pressure; nerve issues, including carpal tunnel syndrome and nerve damage; history of stroke, brain tumor, or brain injury; dizziness, palpitations or fainting; diabetes; current or previous diagnosis of psychiatric condition; use of medication known to alter breathing, blood pressure, or mood; pregnancy or breastfeeding; heavy use of tobacco; frequent migraines; epilepsy; and history of concussion, resulting in loss of consciousness.

The study was approved by the Cardiff University School of Psychology Ethics Committee (EC.18.08.14.5332RA3). All participants gave written informed consent. All methods were performed in accordance with the relevant guidelines and regulations.

#### 2.1.2 Cognitive data acquisition

**Fig. 1.**
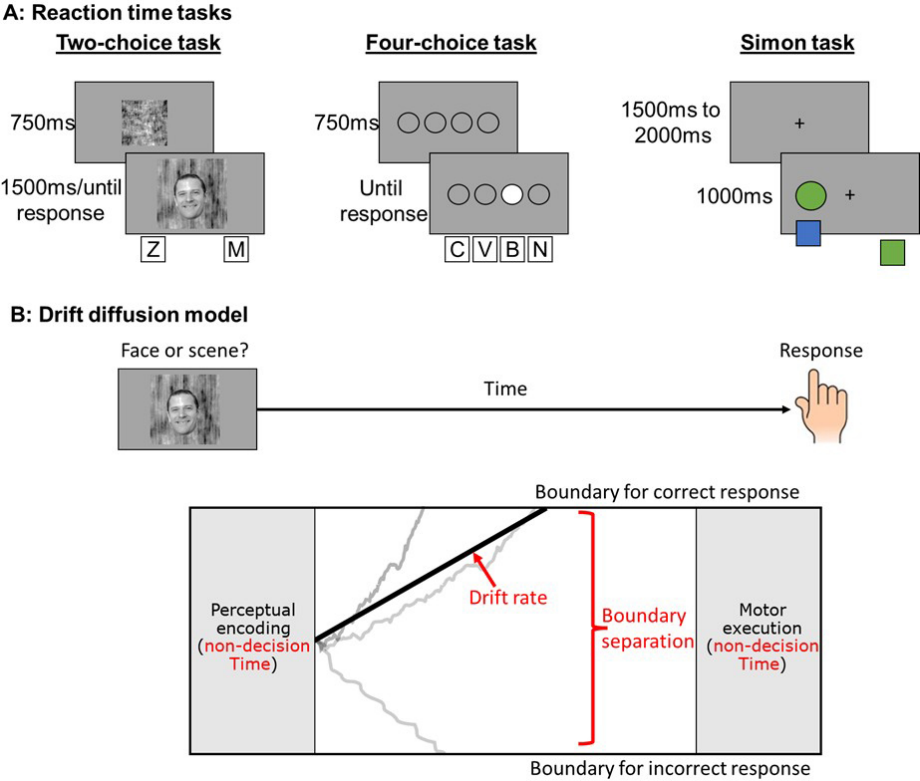
(A) Schematic representation of the choice reaction-time tasks used in the present study. In the two-choice task, participants were instructed to classify a black-and-white picture as either a face or a scene. In the four-choice task, they were asked to press one of four keys to indicate the location of the illuminated circle. In the Simon task, they were instructed to identify the color of the stimulus and ignore its location. The diagram depicts an incongruent trial in the Simon task, where the stimulus (a green circle to the left of central fixation) is presented on the opposite side of the screen to the correct response hand (right). Participants were instructed to respond quickly and accurately in all tasks. (B) A schematic representation of the drift–diffusion model (Ratcliff, 1978) for a face/scene discrimination task. Following stimulus presentation, there is an initial phase of perceptual encoding prior to the decision phase. In the decision phase, the decision process on each trial is represented by the noisy accumulation of evidence to a boundary (gray jagged lines). The drift rate parameter refers to the average rate of evidence accumulation (solid black line), and most closely corresponds to the concept of “processing speed”. When the evidence accumulation process reaches one or the other boundary, a sufficient amount of evidence for that choice has been obtained and the response is initiated. The boundary separation parameter refers to the amount of evidence that a participant requires before triggering a response, with higher values corresponding to a preference for accuracy over speed. Non-decision time is the sum of the perceptual encoding and motor execution phases.

A schematic of the cognitive tasks is shown in Figure 1.

##### 2.1.2.1 Two-choice reaction-time task

Participants were shown black-and-white images of either a face or a scene and were asked to classify them by pressing “z” or “m” on the keyboard, respectively. They were asked to respond as quickly and accurately as possible. Each stimulus was presented until a response was given or for a maximum of 1500 ms, with a 750-ms inter-trial interval. Participants initially completed 10 practice trials followed by 408 experimental trials divided into 3 blocks. Participants received feedback about accuracy in the practice trials but not in the main task. The stimuli were taken from the fLoc functional localizer package (Stigliani et al., 2015).

##### 2.1.2.2 Four-choice reaction-time task

Participants were shown four circles horizontally distributed on the screen. Each circle was unfilled with a black border presented on a gray background. On each trial, one of the circles was filled white, and participants were asked to press the “c”, “v”, “b”, or “n” key corresponding to the location of the filled circle. They were asked to respond as quickly and accurately as possible. Each stimulus was presented until a response was given, with a 750 ms inter-trial interval. Participants completed 8 practice trials followed by 372 experimental trials divided into 3 blocks. The task was based on previous studies that examined white-matter tract characteristics in relation to reaction time (Booth et al., 2019).

##### 2.1.2.3 Simon task

This task (Simon & Rudell, 1967) was included because we recently showed that processing speed contributes substantially to individual differences in performance (Hedge et al., 2022). Participants were shown either a blue or a green circle, which was presented on either the left or right of a central fixation cross. Participants were asked to respond by pressing the left button when the blue circle was presented on the screen and the right button when the green circle was presented. The 50% of trials were congruent, when the circle appears on the same side as the required response (e.g., a blue circle on the left of fixation), and 50% were incongruent, when the location of the circle is on the opposite side to the correct response (e.g., a blue circle on the right of fixation). Participants completed 8 practice trials followed by 400 experimental trials. Stimuli were presented for 1000 ms with an inter-trial interval that varied between 1500 ms and 2000 ms. This task was completed while the participants were seated upright in the MEG scanner, with their head supported by a chin rest to minimize head movement. Further details of the MEG acquisition are not pertinent to this analysis but can be found in the paper describing the data collected in the WAND study (McNabb et al., 2024, 2025).

#### 2.1.3 Neuroimaging data acquisition

The collection and analysis of the MRI data have been described in detail before (Cai et al., 2024) but are presented here for completeness.

T1-weighted images were acquired using magnetization-prepared 180^o^ radio-frequency pulses and rapid gradient-echo (MPRAGE) sequence. MPRAGE sequences were acquired on a Siemens Connectom (300mT/m) 3T scanner, with repetition time (TR) 2300 ms, echo time (TE) 2 ms, field of view (FOV) 256 x 256 x 192 mm, matrix size 256 x 256 x 192, voxel size 1 x 1 x 1 mm, flip angle 9°, inversion time (TI) 857 ms, in-plane acceleration (GRAPPA) factor 2, and phase-encoding direction anterior to posterior (A>>P).

Multi-shell diffusion-weighted MRI data were acquired on the same scanner using protocols described recently for the Microstructural Image Compilation with Repeated Acquisitions (MICRA) dataset (Koller et al., 2021). Data were acquired over 18 minutes using a single-shot spin-echo, echo-planar imaging sequence, in both anterior to posterior (A>>P) and posterior to anterior (P>>A) phase-encoding directions. A>>P data comprised 2 shells of 20 diffusion encoding directions uniformly distributed (Jones et al., 1999) at b = 200 s/mm^2^ and b = 500 s/mm^2^, 1 shell of 30 directions at b = 1200 s/mm^2^ and 3 shells of 61 directions each at b = 2400 s/mm^2^, 4000 s/mm^2^, and 6000 s/mm^2^. Additionally, 2 leading non-diffusion-weighted (b = 0 s/mm^2^) images and 11 non-diffusion-weighted images dispersed throughout (33^rd^ volume and every 20^th^ volume thereafter) were acquired. P>>A data comprised 2 leading non-diffusion-weighted images, 1 shell of 30 directions at b = 1200 s/mm^2^ and a final non-diffusion-weighted image. Data acquisition details for all b-values are as follows: TR 3000 ms, TE 59 ms, FOV 220 x 200 mm in-plane, matrix size 110 x 110 x 66, voxel size 2 x 2 x 2 mm, with in-plane acceleration (GRAPPA) factor 2. The diffusion gradient duration and separation were 7 ms and 24 ms, respectively.

McDESPOT (multicomponent driven equilibrium single pulse observation of T1 and T2) data were acquired using sequences recently described (Deoni et al., 2013), including a T1-weighted 3D spoiled gradient-recalled echo sequence (SPGR), an inversion recovery-prepped spoiled gradient-recalled echo sequence (SPGR-IR), and a steady-state free precession (SSFP) sequence. Data acquisition details were as follows: for SPGR: TR = 4 ms, TE = 1.9 ms, 8 flip angles (3, 4, 5, 6, 7, 9, 13, and 18*^o^*); for SPGR-IR: TR = 4 ms, TE = 1.9 ms, flip angle 5*^o^*, full k-space acquisition in PE and slice directions; for SSFP: TR = 4.54 ms, TE = 2.27 ms, 8 flip angles (10.00, 13.33, 16.67, 20.00, 23.33, 30.00, 43.33, and 60.00*^o^*). For all mcDESPOT data, the phase-encoding direction was A>>P, FOV 220 × 220 × 179 mm^3^, matrix size 128 × 128 × 104, and voxel size 1.72 × 1.72 × 1.72 mm^3^.

### 2.2 Pre-processing and data analysis

#### 2.2.1 Quality control checks for the behavioral data

Participants’ behavioral data for a task were modeled only if their accuracy exceeded 70%. If performance for one task did not meet this criterion, data for that task were imputed as described below in Section 2.2.3. If performance did not meet this criterion for two or more tasks, then the participant’s data were excluded from further analysis.

We removed trials with RTs less than 200 ms and greater than the median plus 3 times the median absolute deviation for each participant in each task (4.5% of trials from participants who met the accuracy criterion).

#### 2.2.2 Cognitive modeling

The drift–diffusion model (Ratcliff, 1978) was fitted to the accuracy coded data from the two-choice reaction-time task and the four-choice reaction-time task using the DMAT toolbox (Vandekerckhove & Tuerlinckx, 2008) in MATLAB. We fit the data for each task and participant independently. For simplicity, we assumed that the drift rate does not vary according to stimulus type (see Supplementary Material S1 for supporting evidence). The starting point of evidence accumulation was fixed to (boundary separation)/2, reflecting a standard assumption that participants have no bias for one response over the others. The full diffusion model also includes parameters capturing trial-to-trial variability in drift rate, starting point, and non-decision time, though we fixed these to zero following expert recommendations to avoid trade-off with the main parameters (Boehm et al., 2018).

Reaction times for correct and incorrect responses were separately binned using percentiles ([10,30,50,70,90,100]). These empirical quantiles were then compared with data simulated from the model, and the deviance (-2 log-likelihood) was minimized using a Nelder–Mead SIMPLEX algorithm (Nelder & Mead, 1965). The result of this process was a set of parameters that most closely simulated our observed results.

The diffusion model for conflict tasks (DMC; (Ulrich et al., 2015)) was fitted to data from the Simon task, using custom code implemented in MATLAB (for previous applications, see Hedge et al. (2019, 2022)). The DMC consists of seven parameters. As with the drift–diffusion model, the main parameters of interest are drift rate, boundary separation, and non-decision time. The DMC captures automatic processing of the irrelevant stimulus location with a scaled gamma function, defined by its amplitude (height) and time-to-peak (scale parameter of the gamma function). The model also includes parameters capturing trial-to-trial variability in starting point and non-decision time. We included these variability parameters in our fits to be consistent with previous applications of the model.

In our approach to fitting the DMC, the deviance was calculated as described for the drift–diffusion model above. Due to the complexity of the model, we repeated the process using multiple starting points. We initially sampled 5000 sets of starting parameters from a uniform distribution, and the best 15 of these were minimized using a Nelder–Mean SIMPLEX that was restarted 3 times to avoid local minima.

#### 2.2.3 Creation of composite measures from drift–diffusion model parameters

Following previous studies using multiple tasks (Haász et al., 2013; Kuznetsova et al., 2016; Penke et al., 2010, 2012), we used a latent variable approach to combine our measures of processing speed (drift rates) into a single latent variable. A common underlying factor is plausible based on previous studies that have shown that drift rate, boundary separation, and non-decision time are correlated across different tasks (Hedge et al., 2022; Lerche et al., 2017). Scores were computed by conducting an exploratory factor analysis and extracting a single factor. Factor scores were computed using the Ten Berge method (Ten Berge, 1999) using the psych package in R (Revelle, 2024). Composite scores for the behavioral measures (mean RT, RT variance, accuracy) were computed in the same way.

A small number of participants did not complete the Simon task. In order to not discard their data, we calculated the full information maximum likelihood (FIML) correlation matrix and used it in the factor analysis in place of the raw data. Factor scores were calculated by imputing missing scores with the mean. We also imputed missing scores for the computation of factor scores if participant’s data for one task were removed for not meeting our accuracy threshold of 70%. A total of 21 missing data points were imputed (20 for the Simon task, 1 for the 4-choice RT task). Data from the Simon task were also imputed for two participants who did not meet the accuracy threshold. These appear to be due to technical issues rather than poor ability, as no responses were recorded for one or both response boxes, so we treat them as missing at random.

#### 2.2.4 Parcellation of cortical and subcortical areas

Cortical reconstruction and volumetric segmentation of the T1-weighted images were performed using the Freesurfer image analysis suite (https://surfer.nmr.mgh.harvard.edu). The technical details of the methods used by the Freesurfer software have been described in other publications (Dale & Sereno, 1993; Dale et al., 1999; Fischl & Dale, 2000; Fischl et al., 1999, 2001, 2002, 2004; Han et al., 2006; Jovicich et al., 2006; Reuter et al., 2010, 2012; Ségonne et al., 2004). The Glasser atlas (Glasser et al., 2016) was used to identify the gray-matter areas that form the nodes of the structural brain networks. The atlas provides 377 cortical and subcortical areas and 2 areas for the left and right cerebellum. The parcellation reconstructed for each participant was overlayed onto the T1-weighted image and was visually inspected to ensure that there was good alignment.

#### 2.2.5 Diffusion-MRI pre-processing

Data were analyzed on a CentOS Linux 7 cluster computing system. The multi-shell diffusion-weighted MRI data were corrected for thermal noise, signal drift, susceptibility-induced distortions, motion and eddy current-induced distortions, gradient non-linearity, and Gibbs ringing artifacts using a combination of in-house pipelines and publicly available software. Using the FMRIB Software Library (FSL version 6.0.3) (Jenkinson et al., 2012), brain extraction was performed (Smith, 2002) and the images were masked to exclude non-brain data. Diffusion MRI noise level estimation and denoising were performed using a Marchenko-Pastur principal component analysis (MP-PCA)-based approach (Veraart, Fieremans, et al., 2016; Veraart, Novikov, et al., 2016). Within-image intensity drift was corrected by fitting the diffusion-weighted MRI data to temporally interspersed non-diffusion-weighted images using in-house code in MATLAB R2017b (MathWorks Inc. Natick, Massachusetts, USA). Slicewise OutLIer Detection (SOLID (Sairanen et al., 2018)) was applied, employing lower and upper thresholds of 3.5 and 10, respectively, using a modified Z-score and a variance-based intensity metric. The susceptibility-induced off-resonance field was estimated from the b = 0 data collected in opposing (A>>P and P>>A) phase-encoding directions using FSL’s topup (Andersson et al., 2003; Smith et al., 2004) and corrected, along with eddy current-induced distortions and subject movements, using FSL’s eddy tool (Andersson & Sotiropoulos, 2016). In-house code was used to correct for gradient non-uniformity distortions in MATLAB R2017b (MathWorks Inc. Natick, Massachusetts, USA). Lastly, Gibbs ringing correction was performed in MRtrix3 using the subvoxel-shifts method (Kellner et al., 2016). The preprocessed images were checked to ascertain lack of any structure in noise maps, absence of significant slice-wise outliers, reduction of movement, good alignment between volumes, lack of implausible signal values, and successful gradient non-uniformity correction. Any participant data that did not satisfy those criteria were excluded from analysis. This resulted in one participant being excluded. The diffusion tensor was calculated from the b = 1200 and 2400 s/mm^2^ diffusion-weighted images.

Preprocessing of mcDESPOT data included brain extraction of T1-weighted MPRAGE data using HD-BET (Isensee et al., 2019) followed by resampling of MPRAGE and masked brain-extracted data to SPGR-IR resolution using Advanced Normalization Tools (Avants et al., 2009). SSFP and SPGR data were corrected for motion using the FMRIB Software Library (FSL version 6.0.1) motion correction tool (Jenkinson et al., 2002) and then SPGR-IR data were registered to the motion-corrected SPGR data using FSL’s linear registration tool (Jenkinson & Smith, 2001). These data were then used to create B1, T1, and B0 maps using functions from QUantitative Imaging Tools (QUIT; Wood, 2018), including driven equilibrium single pulse observation of T1 (DESPOT1) with and without high-speed incorporation, as well as DESPOT2 (Deoni, 2007; Deoni et al., 2003, 2008). Finally, a three-compartment model was used to separate SPGR and SSFP signals into discrete pools representing myelin water and intra-/extra-cellular water or cerebrospinal fluid.

#### 2.2.6 Calculation of fiber orientation distributions and response functions, and tractography

MRtrix3 (Tournier et al., 2019) was used to calculate the response function and the fiber orientations, and to perform anatomically constrained tractography. The response function was calculated using the Dhollander algorithm (Dhollander et al., 2016, 2019). The fiber orientation distributions were calculated using the Multi-Shell-Multi-Tissue constrained spherical deconvolution algorithm (Jeurissen et al., 2014) that allows estimation of multiple crossing fibers within each voxel.

MRtrix3 (Patenaude et al., 2011; Smith et al., 2002, 2012; Zhang et al., 2001) was used to segment anatomical images into cortical and subcortical gray matter, cerebrospinal fluid, white matter, and abnormalities. Anatomical images were co-registered to the diffusion-weighted images using FSL (Jenkinson et al., 2012). The interface between gray matter and white matter was identified using MRTrix3. Anatomically constrained streamline tractography (Smith et al., 2012) was used to generate whole-brain tractograms with the seeds in the gray–white matter interface. All b-value shells were used in the calculation of the tractograms. The minimum and maximum streamline lengths were 30 and 250 mm, respectively, the cutoff was 0.06, and the maximum angle between successive steps was 50 degrees. Thirty million streamlines were generated for each participant. We note that this is a deviation from the 20 million streamlines that were listed in the pre-registration report—this choice was made before the networks were generated because it leads to better reproducibility of the results. The sift2 algorithm (Smith et al., 2015) was used to provide tractograms that have a density of reconstructed connections proportional to the fiber density within each voxel as estimated by the diffusion model. The tractogram reconstructed for each participant was overlayed onto the participant’s fractional anisotropy map and visually inspected to make sure that there is good coverage of the white matter areas and that no streamlines extend into unphysical areas. We note that the fact that each participant’s tractogram contains the same number of streamlines implies that each such streamline represents a specific fraction of the total white matter connectivity for that participant. We return to this in Section 4.

#### 2.2.7 Network generation and edge weight choice

The structural brain networks were constructed by merging the results from the parcellation of the cortical and subcortical areas and the tractography. The nodes were the cortical and subcortical brain areas identified through the Glasser atlas (Glasser et al., 2016), and the edges (connections) were the white-matter tracts connecting those areas.

The number of reconstructed streamlines (NS) is routinely used to assign connection strength in structural brain networks. The NS-weighted connectome has been shown to be good at predicting functional connectivity when used as input in analytical (Goñi et al., 2014; Messaritaki et al., 2021) and machine-learning (Cai et al., 2024) algorithms, and to give good reproducibility in the graph theoretical metrics of structural brain networks (Dimitriadis et al., 2021; Messaritaki et al., 2019). However, the number of streamlines connecting two brain areas depends on the size of the brain areas, with pairs of larger brain areas being assigned more streamlines. Therefore, the number of streamlines itself may not be a good measure of connection strength and must be normalized for the size of the brain areas. A more appropriate measure to use as an edge weight is the number of streamlines divided by the sum of the volumes of the brain areas. We used this measure in this analysis, and we call it ”volume-normalized number of streamlines” (vNS).

The mcDESPOT data allowed us to calculate the myelin water fraction (MWF) in the voxels along the white-matter tracts. Myelin measures could be more functionally relevant than NS-related measures (Cai et al., 2024; Drakesmith et al., 2019; Messaritaki et al., 2021). Given that myelination is known to support fast and efficient signal transport in the brain and to predict electrophysiological functional connectivity (Messaritaki et al., 2021), it could be more relevant when investigating reaction-time tasks. To assess the importance of the two white-matter attributes (vNS and MWF) in explaining the participants’ performance on the reaction-time tasks, we wish to compare the two approaches, and, therefore, calculated structural brain networks in which the edge weights are either vNS or MWF (calculated as the mean across the streamlines of each tract in the tractogram). We note that other studies have used the fractional anisotropy of the diffusion tensor as a proxy for myelination. However, that is less specific to myelin than the MWF, and can be affected by, for example, the orientations of axonal projections within a voxel (Jones et al., 2013).

Following a recent review (Gupta et al., 2021), we selected the areas expected to relate to RT, drift rate, or boundary in our tasks: the frontal eye fields, lateral intraparietal area, striatum, substantia nigra, superior colliculus, pre-frontal cortex (comprising the ventrolateral, ventromedial, and dorsolateral pre-frontal cortex), thalamus, globus pallidus, pre-supplementary motor area, and subthalamic nucleus. Additional areas implicated in reaction-time tasks were selected following Wong et al. (2021) and Hu et al. (2014): the medial frontal cortex, inferior frontal junction, intraparietal sulcus, left cerebellum lobule 6, cerebellum vermis 6, right insula, supplementary motor area, right caudate, and right cuneus. Finally, the following additional areas have been implicated in the Simon task (Cespón et al., 2020): the anterior cingulate cortex, cingulate gyrus, superior frontal gyrus, middle frontal gyrus, inferior frontal gyrus, precentral gyrus, precuneus, inferior parietal lobule, superior parietal lobule, superior temporal gyrus, and middle temporal gyrus. We used these areas as nodes of the task-specific network. In the Glasser atlas, this amounts to 193 regions of interest.

#### 2.2.8 Selection of graph theoretical metrics

The characteristic path length, global efficiency, and navigation efficiency were calculated, based on relationships of these metrics with reaction-time measures that have been found previously (Caeyenberghs et al., 2014; Imms et al., 2021; Reijmer et al., 2013). However, we inspected our data and found that global efficiency and navigation efficiency were extremely highly correlated, as expected, since they are derived from common underlying metrics (r > 0.9, as shown in the Supplementary Material S2). Path length was also highly correlated with the efficiency metrics (r > 0.8). This is in congruence with the fact that the global efficiency is related to the average of the inverse path lengths between nodes. We, therefore, selected just one metric for analysis, focusing on global efficiency as the more widely understood measure. We note that this approach is described as an amendment to our pre-registration (Messaritaki et al., 2023), where all three graph theoretical metrics were intended to be included as regressors in a general linear model, which could not be done due to the multi-collinearity.

#### 2.2.9 Outliers

In the final stage of pre-processing, we removed multivariate outliers based on a robust Mahalanobis distance using a cutoff of α = .001 (Leys et al., 2018). This was done separately for each graph theoretical-metric/cognitive-measure pair.

### 2.3 Statistical analysis

#### 2.3.1 Main analysis

Our first aim was to understand whether processing speed is related to network characteristics at whole-brain level and in a task-specific subnetwork. We used linear regression to test the association between global efficiency (predictor) and composite drift rate (outcome). We conducted the analysis once using the global efficiency of the task-specific network and once using the global efficiency of the whole brain.

We repeated these analyses for the other behavioral metrics as the outcome variable in place of drift rate: composite boundary separation; composite non-decision time, composite mean RT, composite variability in RT, and composite accuracy.

To test whether the task-specific network is preferentially associated with behavioral measures, we compared the task-specific network with null-model subnetworks as follows: We randomly selected 10,000 subnetworks of 193 brain areas each (i.e., the same number of brain areas as our task-specific subnetwork) with the combination of those brain areas being unlinked to reaction-time tasks (task-unrelated subnetworks). We calculated the global efficiency of each of the random, task-unrelated subnetworks and repeated the above-mentioned linear regression analysis for each of them. This resulted in a range of values for the regression coefficients, on which we fit a (Gaussian) distribution. The values of the regression coefficients calculated from the analysis for the task-specific subnetwork were compared against those distributions to evaluate the likelihood that the observed regression coefficients could have resulted by any random subnetwork. We used a z-test to calculate the likelihood of observing the task-specific subnetwork regression coefficient from the distribution of those from the random subnetworks.

#### 2.3.2 Control analysis: Age dependence

Processing speed and network characteristics vary with age (Lu et al., 2011). To inform any associations found between global efficiency and behavioral measures, we performed control analyses with age included as a covariate in the regression. We also conducted a mediation analysis in cases where we observed a significant association between a cognitive measure and WM characteristics in the simple regression, but the association was no longer significant when age was included as a covariate.

## 3 Results

**Fig. 2.**
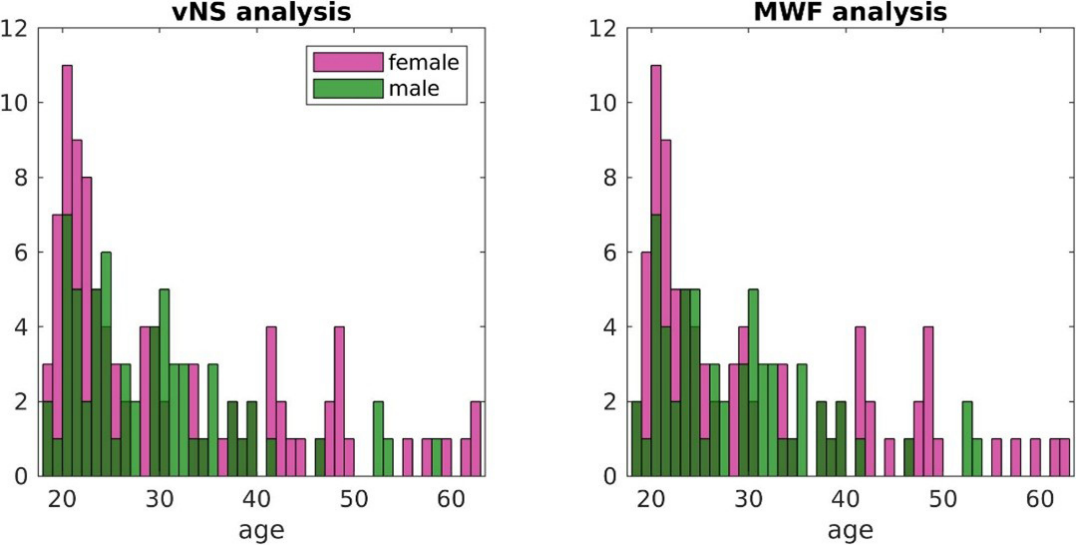
Age (years) and gender of the participants. Left: vNS analysis; right: MWF analysis.

Following data quality checks described above, there were 159 participants (94 female) left for the volume-normalized number-of-streamlines (vNS) analysis, 146 (85 female) of whom were also appropriate for the myelin–water-fraction (MWF) analysis. The age distributions of the participants are shown in Figure 2.

### 3.1 Outliers

The number of participants included in the final analysis, after outlier removal, is given in Table 1. The final sample sizes were lower for the MWF analyses relative to the vNS analyses, but the impact on statistical power is small. For example, sensitivity power analyses conducted for the drift rate analysis give 80% power to detect correlations of r = .22 in the vNS analysis (N = 159) and r = .23 in the MWF analysis (N = 144). The lowest sample size in any analysis was N = 119, which gives 80% power to detect r = .25.

**Table 1.** Number of participants included in analysis after outlier exclusion.

Edge weight	Accuracy	Mean RT	SD RT	Drift rate	Boundary separation	Non-decision
vNS	154	155	157	159	131	141
MWF	142	144	144	145	119	130

Note: Outlier analysis was performed on N = 159 for vNS and N = 146 for MWF. Data points were considered outliers by a threshold of *p* < .001.

vNS = volume-normalized number of streamlines; MWF = myelin water fraction; SD = standard deviation.

### 3.2 Behavioral performance and composite scores

Descriptive statistics for raw behavioral performance and diffusion model parameters are reported in Table 2 along with the correlations between measures. Performance levels were typical of choice reaction-time tasks to which the diffusion model is commonly applied, with mean reaction times less than 1000 ms and accuracy over 90% (Lerche et al., 2017; Ratcliff & McKoon, 2008). For the raw behavioral measures (accuracy and reaction time) and drift rate, between-task correlations were consistently positive and ranging from r = .26 to r = .61. Between-task correlations for boundary separation and non-decision time were noticeably lower, particularly between the four-choice and Simon tasks. These lower correlations for the model parameters are also reflected in the fit statistics for the factor analysis (Table 3). Common rules of thumb for evaluating the appropriateness of a factor analysis include eigenvalues greater than 1, KMO values greater than .6, and a significant Bartlett’s test. These criteria are met for all the variables, except for the KMO values for boundary separation and non-decision time. Critically, the conventional criteria are met for drift rate, which is our main focus. However, the proportion of variance explained by the factors is also relatively low for the model parameters. Our interpretation of these results is that there is common variance in the behavioral variables and model parameters, which is consistent with the assumptions of our pre-registered analysis plan and so we base our conclusions on our analysis using the composite scores. We also conduct an exploratory analysis in which we use the individual task variables as predictors in place of the composite scores (see Section 3.5.2).

**Table 2.** Pearson’s r correlations, means, and standard deviations for behavioral measures and diffusion model parameters.

		Accuracy (%)			Mean reaction time (ms)			SD reaction time (ms)			Drift rate			Boundary separation			Non-decision time		
Variable	Task	Two	Four	Simon	Two	Four	Simon	Two	Four	Simon	Two	Four	Simon	Two	Four	Simon	Two	Four	Simon
	Mean	95.3	94.7	94.9	445	382	453	72	65	92	0.60	0.43	51.6	0.32	0.16	72.43	205	229	360
	SD	3.3	3.4	3.9	56	74	54	19	24	24	0.25	0.18	20.3	0.2	0.16	11.52	90	74	49
Accuracy	Four	**0.54**																	
	Simon	**0.40**	**0.29**																
Mean RT	Two	0.07	0.20	-0.16															
	Four	0.03	0.39	-0.09	**0.58**														
	Simon	-0.13	0.10	-0.23	**0.61**	**0.35**													
SD RT	Two	-0.25	-0.07	-0.30	0.72	0.27	0.57												
	Four	-0.03	0.24	-0.20	0.50	0.82	0.40	**0.37**											
	Simon	-0.20	-0.03	-0.45	0.39	0.13	0.81	**0.56**	**0.29**										
Drift rate	Two	0.72	0.54	0.39	-0.10	0.07	-0.21	-0.46	-0.01	-0.30									
	Four	0.28	0.62	0.32	0.12	0.13	-0.06	-0.07	-0.13	-0.19	**0.29**								
	Simon	0.22	0.30	0.48	-0.04	0.19	-0.01	-0.15	0.09	-0.24	**0.26**	**0.30**							
Boundary separation	Two	0.64	0.51	0.24	0.22	0.17	0.06	-0.05	0.16	-0.03	0.85	0.27	0.19						
	Four	0.16	0.54	0.22	0.25	0.39	0.12	0.13	0.27	-0.04	0.15	0.82	0.32	**0.24**					
	Simon	0.18	0.20	0.32	-0.04	0.02	-0.01	-0.07	-0.10	-0.17	0.09	0.25	0.31	**-0.01**	**0.19**				
Non-decision	Two	-0.52	-0.38	-0.22	0.06	0.06	0.05	0.04	-0.03	0.02	-0.71	-0.18	-0.16	-0.90	-0.18	0.02			
	Four	-0.13	-0.26	-0.21	0.10	0.21	0.04	-0.05	0.04	0.02	-0.08	-0.60	-0.17	-0.17	-0.75	-0.12	**0.29**		
	Simon	0.04	-0.06	0.08	0.08	-0.02	0.02	-0.01	-0.03	0.07	-0.05	-0.08	-0.12	-0.01	-0.10	-0.01	**0.04**	**0.08**	

Note: After exclusions based on behavioral performance: Two-choice N = 160, four-choice N = 159, Simon N = 138. Triads in bold correspond to variables on which the factor analyses were conducted. The drift rate and boundary separation estimates for the Simon task are on a different scale to those for the two- and four-choice tasks, so means and standard deviations should not be directly compared.

SD = Standard deviation.

**Table 3.** Factor loadings and fit statistics for choice reaction-time tasks.

Variable	Mean RT	SD RT	Accuracy	Drift rate	Boundary	Non-decision time
Two-choice	1.00	0.88	0.86	0.50	0.24	0.39
Four-choice	0.58	0.42	0.63	0.59	1.00	0.74
Simon	0.61	0.62	0.46	0.50	0.20	0.11
Eigenvalue	2.02	1.79	1.83	1.56	1.31	1.31
Proportion variance	0.57	0.44	0.45	0.28	0.36	0.24
KMO	0.60	0.60	0.61	0.62	0.48	0.51
Bartlett χ^2^	137.00	78.93	83.48	33.21	16.05	14.86
Bartlett *p*-value	<.001	<.001	<.001	<.001	0.001	0.002

### 3.3 Associations between cognitive measures and global efficiency

**Fig. 3.**
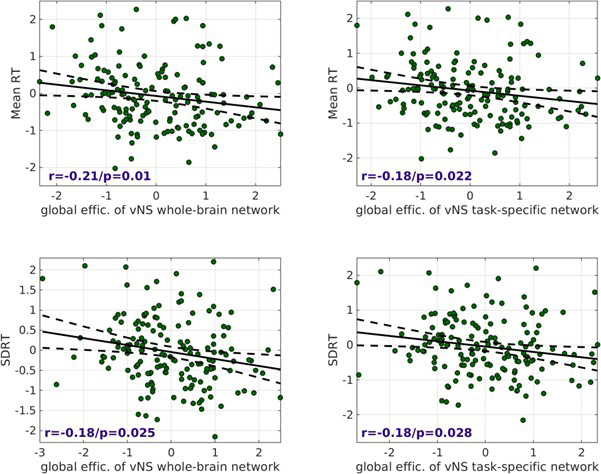
Scatter plots showing the relationships between global efficiency and mean/variability of RT, for the vNS-weighted whole-brain and task-specific networks. The solid line indicates the best-fit line resulting from the general linear model, and the dashed lines are the 95% confidence intervals. The corresponding correlation coefficients and *p*-values are shown.

We did not observe any significant associations between the model parameters and global efficiency (see Table 4). In a partial replication of previous findings, we found that global efficiency was slightly (i.e., with small effect size) negatively correlated with both mean reaction time and reaction-time variability (Fig. 3). This was only observed when global efficiency was calculated from the vNS-weighted networks data and not the MWF-weighted ones (see Section 4 for a possible explanation). We focus on the vNS results for the rest of this section. No significant associations were observed with accuracy.

**Table 4.** Standardized estimates from simple linear regressions predicting each cognitive outcome by global efficiency.

	Number of streamlines by volume (vNS)				Myelin water fraction (MWF)			
	Whole-brain network		Task-specific network		Whole-brain network		Task-specific network	
Outcome	β	*p*	β	*p*	β	*p*	β	*p*
Drift rate	0.07	.401	0.07	.390	0.06	.442	0.06	.443
Boundary	-0.02	.786	-0.04	.673	0.07	.479	0.06	.501
Non-decision	-0.02	.851	-0.03	.723	-0.11	.223	-0.15	.095
Mean RT	**-0.18**	**.025**	**-0.18**	**.028**	0.05	.568	0.04	.640
SD RT	**-0.21**	**.010**	**-0.18**	**.022**	0.10	.216	0.11	.179
Accuracy	-0.03	.705	-0.04	.651	0.04	.619	0.02	.783

Note: Separate regressions were conducted for each outcome. See Table 1 for Ns. Each model had a single predictor, therefore, the β values are equivalent to Pearson’s correlation coefficients r. In line with Cohen’s (1988) criteria for correlations, β values of 0.1 and 0.3 correspond to small and medium effect sizes, respectively. Significant associations (*p *< .05, uncorrected) are highlighted in bold. We base our interpretations on these uncorrected *p*-values, though we note that none would survive a Bonferroni correction for multiple comparisons (adjusted α = .002 based on 24 effects reported in the table).

**Fig. 4.**
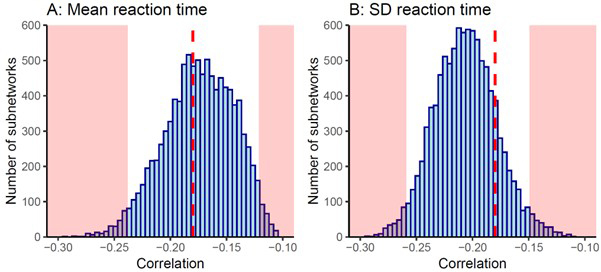
Random-subnetwork analysis for the task-specific subnetwork. Histogram of the correlation coefficients between global efficiency and mean RT (A) and RT variability (B), for the 10,000 non-task-specific (random) subnetworks. The vertical red line indicates the observed correlation for the task-specific subnetwork. The red shaded areas represent the 2.5^th^ and 97.5^th^ percentiles of the random-subnetwork distribution. The observed correlation for the task-specific subnetwork would need to be within the red-shaded region(s) to be considered statistically distinct from random networks with the same number of nodes (*p* < .05, two-tailed).

The random-subnetwork testing showed that the correlations observed in the task-specific subnetwork did not statistically differ from the correlations obtained from subnetworks with the same number of randomly selected nodes, for both mean RT (*p* = .95) and SD RT (*p* = .38; see Fig. 4). These results indicate that the observed relationships are more likely to be due to whole-brain characteristics of the white-matter organization, rather than those of the task-specific subnetwork. The two-tailed *p*-values were calculated by first mean centering the observed and random-subnetwork correlations using the mean of the random-subnetwork distribution. The *p*-value was then calculated as the proportion of the absolute random-correlation values that were equal to or greater than the observed correlation in the task-specific network. Note that neither of the random distributions in Figure 4 include r = 0, which speaks of the robustness of the finding at the whole-brain level. Global efficiency is negatively correlated with both mean RT and SD RT no matter which subnetwork we examine.

### 3.4 The contribution of age

**Fig. 5.**
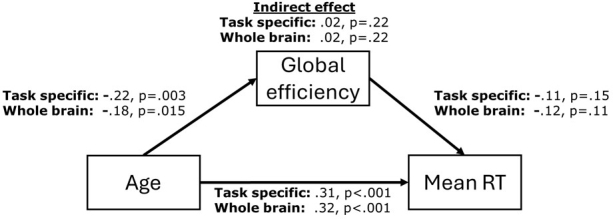
Standardized regression coefficients for the relationship between age and mean reaction time as mediated by global efficiency.

We re-ran each of the regressions reported in Table 4 with age as an additional predictor (Table 5). Age was a statistically significant predictor for mean RT and accuracy, as expected (and the model parameters that depend on them), but not the variability of RT. Notably, global efficiency remains a significant predictor of RT variability when controlling for age, but there is no longer a significant association between global efficiency and mean RT. Note, however, that the regression coefficients show a similarly small negative association in all models (β’s ranging from –0.11 to –0.18, see Table 4), the different *p* values should not be interpreted too categorically. In sum, age correlates reliably with both white-matter metrics and behavior, and appears to be a marker for a proportion of the association between global efficiency and mean RT. In other words, the long-known association between age and RT may be partially mediated by white-matter changes captured by global efficiency. Per our pre-registration, we conducted a mediation analysis to evaluate whether global efficiency mediated the association between age and mean RT (Fig. 5). The analysis was conducted using the lavaan package in R (Rosseel, 2012). The indirect effect was not statistically significant in either the whole-brain or task-specific networks (*p* = .22).

**Table 5.** Standardized estimates from multiple linear regressions predicting each cognitive outcome by global efficiency and age.

		Number of streamlines by volume				Myelin water fraction			
		Whole-brain network		Task-specific network		Whole-brain network		Task-specific network	
Outcome	Predictor	β	*p*	β	*p*	β	*p*	β	*p*
Drift rate	Global Eff.	0.13	.101	0.14	.066	0.05	0.492	0.05	0.514
	Age	**0.34**	**<.001**	**0.35**	**<.001**	**0.31**	**<.001**	**0.31**	**<.001**
Boundary Separation	Global Eff.	0.01	.948	0.01	.863	0.06	0.505	0.05	0.566
	Age	**0.39**	**<.001**	**0.39**	**<.001**	**0.39**	**<.001**	**0.39**	**<.001**
Non-decision Time	Global Eff.	-0.03	.729	-0.05	.571	-0.11	0.232	-0.14	0.102
	Age	-0.13	.125	-0.13	.116	-0.09	0.293	-0.09	0.303
Mean Reaction time	Global Eff.	-0.12	.111	-0.11	.168	0.04	0.594	0.03	0.688
	Age	**0.32**	**<.001**	**0.31**	**<.001**	**0.31**	**<.001**	**0.31**	**<.001**
SD of Reaction time	Global Eff.	**-0.20**	**.014**	**-0.17**	**.032**	0.10	0.220	0.11	0.184
	Age	0.05	.559	0.04	.590	0.03	0.679	0.03	0.686
Accuracy	Global Eff.	0.02	.782	0.03	.697	0.04	0.654	0.02	0.845
	Age	**0.35**	**<.001**	**0.35**	**<.001**	**0.34**	**<.001**	**0.34**	**<.001**

Note: Separate regressions were conducted for each outcome. See Table 1 for Ns. Significant associations (*p* < .05, uncorrected) are highlighted in bold. For all models where age is a statistically significant predictor, a model comparison with the equivalent model without age included (see Table 4) indicated a significantly improved fit for the model including age (*p* < .001 for all significant model comparisons). No result survived Bonferroni correction (α = .002).

### 3.5 Exploratory analyses and sanity checks

In addition to our pre-registered analyses described above, we and reviewers identified additional questions that could be asked of our data. For readability, we summarize the conclusions in the main text and include relevant tables in Supplementary Materials.

#### 3.5.1. Parameter recovery for drift–diffusion model parameters

Parameter recovery simulations were performed for the drift–diffusion model fits to determine whether individual differences in the parameters can be identified. These consisted of simulating data from the best fitting values for each participant and then fitting the model to these simulated data. Correlations between simulated and recovered parameters were acceptable for the three parameters of interest (drift rate, boundary separation, and non-decision time), ranging from r = .69 to r = .98 (see Supplementary Material S3).

#### 3.5.2 Individual tasks as predictors instead of composite scores

Our factor analysis fit statistics (Table 3) indicate that there is common variance in our cognitive measures of interest. However, there is also task-specific variance that our pre-registered analysis using composite scores does not account for. As a sanity check, we ran exploratory multiple regressions in which we used the individual task variables as independent predictors and global efficiency as the outcome variable (Supplementary Material S4). These exploratory analyses did not yield a consistent pattern of associations between global efficiency and any behavioral measure or model parameter.

#### 3.5.3. Estimating the drift–diffusion model parameters with a simpler model

In our main analysis, we used the DMAT toolbox to estimate the drift–diffusion model parameters for the two-choice and four-choice tasks, and the DMC to estimate parameters from the Simon task. These versions of the models use optimization algorithms to identify the parameter values that provide the best fit to quantiles of correct and error reaction-time distributions. It has been noted that a simplified version of the drift–diffusion model, the EZ-diffusion model (Wagenmakers et al., 2007), sometimes outperforms more complex versions of the model when it comes to parameter recovery and the detection of experimental effects (Lerche et al., 2017; van Ravenzwaaij et al., 2017). To evaluate whether using a simpler form of the model changes our conclusions, we re-ran our main analysis using the factor scores (Table 3) and individual tasks (Supplementary Material S4) using estimates from the EZ-diffusion model (Supplementary Material S5). This did not lead to different conclusions. When using the composite scores, we observed no significant associations between global efficiency and drift rate, boundary separation or non-decision time. When using the individual tasks as predictors, again this did not yield a consistent pattern of associations between global efficiency and any model parameter.

#### 3.5.4 Age as a moderating variable

A reviewer suggested that we consider age as a moderating variable in the association between global efficiency and cognitive variables. To examine this, we ran multiple linear regression models with the cognitive variables as the outcome and global efficiency, age, and the global efficiency x age interaction as predictors (Supplementary Material S6). We observed one statistically significant interaction (uncorrected), in the analysis of the MWF data when drift rate was the outcome. Plotting this interaction revealed that global efficiency was positively correlated with drift rate in younger adults in our sample but negatively correlated in older adults. Though this interaction is potentially of theoretical interest, we are cautious of overinterpretation. We had no hypotheses about interaction effects and it was not seen in the vNS data.

#### 3.5.5 Mediation model for reaction-time variability

**Fig. 6.**
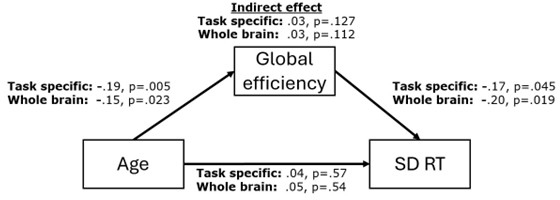
Standardized regression coefficients for the relationship between age and variability of the mean reaction time as mediated by global efficiency.

In our pre-registered analysis plan, we stated that we would run the mediation analysis if we observed a significant association between a cognitive measure and white-matter characteristics in the simple regression, but the association was no longer significant when age was included as a covariate. Mean reaction time was the only variable that met this criterion. For completeness, we also conducted a mediation analysis for SDRT, which was significantly associated with global efficiency in both the simple (Table 4) and multiple regression (Table 5). The indirect effect was not statistically significant in either the whole-brain (*p* = .11) or task-specific networks (*p* = .13) (Fig. 6 and Supplementary Material S7).

## 4 Discussion

Our study represents the largest scale investigation to date of brain microstructure using ultra-strong gradient MRI, with preregistered analyses. We used high-gradient diffusion to enhance the fidelity of both microstructural metrics and tractography reconstructions, giving us the best possible chance of detecting associations between behavioral measures and network characteristics. Our findings replicate previous evidence that mean reaction time and reaction-time variability are associated with white-matter characteristics (Haász et al., 2013; Kievit et al., 2016; Kuznetsova et al., 2016; Penke et al., 2010, 2012), albeit the effect sizes we observe are relatively small. This reduction in effect size compared with previous smaller scale reports is consistent with findings in pre-registered studies in other topics and has been explained via reporting biases whereby non-registered studies with non-significant results are less likely to reach publication (Kvarven et al., 2020; Schäfer & Schwarz, 2019). When we applied a theoretically informed model of speeded choice to extract a “processing speed” parameter, we found that neither drift rate nor any other parameters from the models were associated with the white matter. We will return to this later in Section 4.

### 4.1 Processing speed and white matter

Our observations that the mean and SD of reaction times are associated with white-matter characteristics are consistent with the proposal that the efficient communication of neural signals underlies cognitive processing (Eysenck, 1982; Jung & Haier, 2007). This is also consistent with the “neural noise hypothesis” (Kail, 1997; McCormick et al., 2023), where it is proposed that demyelination in aging and in pathological conditions such as multiple sclerosis decreases the signal-to-noise ratio of neural signals. We found that this correlation is statistically indistinguishable when comparing global efficiency calculated from a task-specific subnetwork with the whole brain. The parieto-frontal theory of general intelligence assumes that efficient communication between specific regions underlies individual differences in complex cognitive processes (Jung & Haier, 2007). However, our findings do not contradict this theory, as it does not assume that white-matter characteristics are independent across the brain. We return to the role of the task-specific subnetwork later on. Our findings also agree with the results of studies of functional brain networks from both fMRI (van den Heuvel et al., 2009) and EEG (Langer et al., 2012) recordings, which have shown a link between functional network efficiency and intelligence or intellectual performance, both of which are inversely correlated to RT variability.

The effect sizes that we observe, r = -.18 and r = -.21 for mean RT and SD RT, respectively, are small according to traditional heuristics (Cohen, 1988). However, they are close to the median of r = .19 observed in reviews of the effect sizes observed in psychology (Gignac & Szodorai, 2016). Recent examinations of large neuroimaging consortia datasets have also noted that univariate correlations between brain and behavior are typically small (Marek et al., 2022). Large effect sizes between white-matter characteristics and processing speed have only been reported with small cohorts (e.g. N = 12 (Tuch et al., 2005)). Estimates of the association between processing speed and white-matter characteristics from other larger cohorts are closer to those we observed here (Booth et al., 2019; Haász et al., 2013; Kievit et al., 2016; Kuznetsova et al., 2016; McCormick et al., 2023; Penke et al., 2010, 2012). Analysis of the Lothian Birth Cohort, an older adult sample (Penke et al., 2012), observed a standardized estimate of β = -.19 between a processing speed factor comprising 3 tasks and a fractional anisotropy factor comprising 12 tracts (higher fractional anisotropy factor was related to higher processing speed; see also Penke et al. (2010) for an earlier analysis of the same cohort). Another recent analysis (McCormick et al., 2023) reported negative associations between fractional anisotropy in the anterior thalamic radiations and latent factors representing mean RT (β = -.32) and RT variability (β = -.33). The authors also report similar associations in 9 out of the 10 tracts examined. The heterogeneity in methods makes direct comparisons between these studies difficult—they vary in the age of the cohort, the tasks used, and where and how white-matter characteristics are measured. However, taken together, they paint a picture of a modest association between white-matter characteristics and both reaction time and its variability.

We observed no significant associations between accuracy and white-matter characteristics in our data. Reaction-time variables are typically the focus in the processing *speed* literature, though speed and accuracy are linked both conceptually and in evidence-accumulation models (Ratcliff & Rouder, 1998; Wickelgren, 1977). If the global efficiency of white-matter networks does underlie efficient cognitive processing, then we would expect that to manifest in performance that is both faster and more accurate. However, the relationship between speed and accuracy is not linear, so they may not be equally sensitive to individual differences in white-matter characteristics.

### 4.2 Number of streamlines by volume vs. myelin water fraction

In our main analysis (Table 4), we observed a significant association between the RT variability and the vNS-based global efficiency, but not the MWF-based global efficiency. One likely reason is that the variance across individuals in the MWF measure was too small (coefficient of variation = 0.04 compared with ~0.2 for vNS). A key challenge for myelin-based studies of individual differences is the relative homogeneity of myelin in healthy individuals. Even if myelin binding properties vary between individuals, the fraction of water trapped within the layers may not change substantially (although note that there was sufficient variance to show association between global efficiency and age in the MWF-based measure).

In contrast, streamline count is a fundamentally different measure, influenced by factors such as the distance between regions (which leads to a natural drop-off for regions that are further apart), curvature, anisotropy, signal-to-noise ratio (SNR), and bundle thickness—though the volume-normalized approach used here partially accounts for this. Streamline count, therefore, integrates multiple factors relevant not just to conduction speed but also to conduction delay, including length, geometry, and connectivity patterns, resulting in far greater variance in volume-normalized streamline count (vNS) than MWF. Moreover, axon diameter plays a larger role than myelin in determining conduction velocity (Drakesmith et al., 2019). Therefore, it is fully consistent with our understanding of the measures that vNS should be more likely than MWF to show correlations with behavioral individual differences in healthy participants.

### 4.3 Whole-brain vs. task-specific networks

We observed significant correlations for mean RT and SDRT in both the task-specific and whole-brain networks, and the correlations were numerically equivalent or lower for the task-specific subnetwork than for the whole-brain network. We calculated the correlations across participants between the global efficiency of the whole-brain and task-specific network and found that it was 0.98 for the vNS-weighted networks and 0.96 for the MWF-weighted networks. This, along with the fact that the random-subnetwork analysis showed that the correlations for the task-specific subnetwork are within the distribution correlations for subnetworks consisting of the same number of non-task-specific nodes, points to the fact that, based on our analysis, it is not possible to distinguish the role of a task-specific subnetwork from brain-wide properties.

### 4.4 The role of age

Age often correlates with mean RT and accuracy and does so in our data. When age was entered as an additional regressor, a significant association with global efficiency remained for SDRT, but not for mean RT. However, we caution against a strong interpretation of this. The initial correlations were relatively small, and both are reduced to some degree by allowing age to explain some variance. The fact that results fall on either side of a traditional *p* value criterion should not be over-interpreted.

It has been noted that chronological age is not a psychological variable in itself, so associations with age provide additional information but do not *explain* the association between network characteristics and RT (Wohlwill, 1970). Instead, age is a proxy for a multitude of brain-related variables that change over time, of which white-matter characteristics is one. One possibility is that the well-known correlation between age and RT could be mediated by the measured network characteristics, but indirect effects based on small effect sizes are difficult to detect and we did not find evidence for this in our data.

In our analysis, age was positively associated with reaction time, accuracy, drift rate, and boundary separation. This indicates an age-related slowing, and points to the fact that older adults are more cautious and more accurate. These results are a replication of what has been previously found.

Previous studies have tested whether neuroimaging measures mediate the association between age and components of fluid cognition, including processing speed, executive functioning, and memory (Hedden et al., 2016; Madden et al., 2020; Merenstein et al., 2023), though variation in design and analysis choices makes it difficult to draw a clear conclusion about processing speed specifically. Hedden et al. (2016) found that whole-brain FA and striatal volume fully mediated the association between age and a composite processing speed factor in a sample of older adults. Their processing speed measures included two pen-and-paper neuropsychological tests (trail-making and digit-symbol substitution) alongside a choice RT measure, in contrast to our study that focuses on choice RT. Madden et al. (2020) did not test the mediation effect for processing speed because they did not observe a correlation with age after controlling for performance on the other cognitive tasks and gender. They did find that a functional graph theoretical measure, system segregation, fully mediated the association between age and executive functioning measures (digit symbol substitution, verbal fluency, Stroop interference), but structural measures did not. No significant mediation effects were observed when an overall fluid cognition composite was used as the outcome. Merenstein et al. (2023) also did not report a mediation model for processing speed specifically, but they did for an overall fluid cognition composite (speed, executive functioning, and memory). They found partial mediation effects for mean diffusivity in the ventral attention network, dorsal attention within-network connectivity, and subcortical between-network connectivity. In summary, multiple studies have been able to partially account for the association between age and cognition using measures of brain structure and function. However, results vary in terms of the magnitude of the mediation effect, the imaging variables used, and the cognitive domain in which a mediation is observed.

### 4.5 Why correlations in behavioral measures are not reflected in decision model parameters

It may seem surprising that the correlations we observe in reaction time are not reflected in any of the decision model parameters. However, this is not unprecedented in the literature. Previous findings using evidence-accumulation models are mixed, with some observing correlations in, for example, drift rate (Imms et al., 2021; Madden et al., 2009) and others not (Karahan et al., 2019). One methodological limitation of previous modeling studies was the use of single tasks rather than composite measures derived from a latent factor representing multiple tasks. These composite/factor scores have desirable psychometric properties such as improved reliability (Eisenberg et al., 2019), which aid our ability to detect associations. Nevertheless, when using composite measures for both reaction time and model parameters, we observed statistically significant correlations in the former and not in the latter.

To explain this, we can consider how the diffusion model parameters capture individual differences in practice. Though drift rate is conceptually linked to “processing speed” (Myers et al., 2022; Ratcliff et al., 2004; van Ravenzwaaij et al., 2011), empirically the parameter normally shows stronger correlations with accuracy and RT variability than with mean RT (Table 2; see also Matzke & Wagenmakers (2009); Ratcliff et al. (2015)). Given that we observe no significant correlations between network characteristics and accuracy, and drift rate is most strongly linked to accuracy in our data, the discrepancy between the results for drift rate and RT is less surprising.

However, boundary separation and non-decision time are generally correlated with RT (Matzke & Wagenmakers, 2009; Ratcliff et al., 2015), but here these parameters did not mirror the correlations shown by mean RT either with global efficiency or across tasks. This may be because fitting the models can be susceptible to noise, causing parameters to trade off. For some individuals, a higher mean RT might be absorbed more by boundary separation, whereas for others it will be captured by longer non-decision time due to idiosyncrasies in the behavioral distributions rather than because the longer RTs have genuinely different sources in the two people. For example, fit non-decision time often takes values that are implausible for biologically sensory and motor processes, and this has knock-on consequences for the estimation of other parameters (Bompas et al., 2025). Such challenges are exacerbated in, but not restricted to, datasets where there are few errors or the overall trial numbers are low. We administered a relatively large number of trials in our tasks (372 to 408), based on simulations (Lerche et al., 2017) showing that 283 trials were optimal for estimating the 3 main parameters when error rates were at least 4% and 314 were optimal for estimating drift rate (and non-decision time) with fewer than 4% errors. The *average* error rates in our tasks were ~5%, so we are consistent with these recommendations, but parameter estimation may have been compromised for participants close to ceiling accuracy. The impact of this for our analysis is that it would add noise. In this light, it is worth noting that that the inter-task correlations for mean reaction time (r = .35 to r = .61) and SD reaction time (r = .29 to r = .56) were generally higher than for the model parameters, with drift rate being the only parameter showing consistent positive correlations (r = .26 to r = .30; see Table 2). These lower correlations were also reflected in the factor analysis fit statistics. The KMO values for boundary separation and non-decision time were modest, and the variance explained by the composite scores for the model parameters was relatively low (24%–36%) compared with the composite scores for the behavioral measures (44%–57%). This is to be expected if the model parameters are noisier. Indeed, previous studies have shown that the test–retest reliability of drift rate is consistently comparable with mean RT, whereas non-decision time is typically less reliable (Enkavi et al., 2019; Hedge et al., 2019; Lerche & Voss, 2017). We are less likely to detect correlations with global efficiency where behavioral composite scores do not reflect common variance across tasks. However, we also saw no consistent associations between model parameters and global efficiency when the individual task parameters were used as predictors in place of the composite scores (Supplementary Material S4).

Overall, our results suggest caution when assuming that drift rate should be the primary variable for researchers interested in the neural mechanisms of *processing speed*, converging with other recent reports that decision model parameters should not be assumed to reflect the constructs they are conceptually associated with (Bompas et al., 2025). They may be better viewed as composite parameters that relate RT to accuracy, rather than cleanly dissecting cognitive processes.

A final possibility is that drift rate does successfully extract a psychologically meaningful construct, but it is not the one most correlated with global efficiency of large brain networks. It has been argued that drift rates can be interpreted as a measure of the ability to “rapidly and selectively extract goal-relevant information” (Weigard et al., 2021) (pg. 2), with the emphasis on selection and goal-relevance linking the parameter to the constructs of selective attention and executive functioning (Weigard & Sripada, 2021). Based on this, we would expect drift rate to differentiate between individuals most effectively in a relatively difficult task. In contrast, some theorists have historically encouraged the use of simple tasks that produce low error rates to capture processing speed (Salthouse, 1993). Future work to understand the neural correlates of evidence accumulation model parameters may benefit from additionally using more difficult tasks, in a departure from how processing speed is typically measured.

### 4.6 Caveats, limitations, and future directions

The participants in this study were predominantly 18–30 years old, with only 34% of them in the wider age range of 31–63 years. Additionally, as is the case in many similar studies, the recruitment methods bias toward people who are likely educated for longer than average. For that reason, our results may be missing effects that are present in the general population.

As mentioned in Section 2.2.6, the fact that each participant’s tractogram contains the same number of streamlines implies that each such streamline represents a fraction of the total white-matter connectivity for that participant. This is in accordance with what is done in similar studies and should be kept in mind in the interpretation of the results. Different methods for constructing connectivity matrices from tractograms are employed in the literature. We note that our use of sift2 is equivalent to the normalization proposed by Smith et al. (2022), since multiplication by the additional factors in equation (4) of that paper would make no difference in our regression results, because those factors are the same across participants.

The results we reported were without any multiple-comparison correction and the small effect sizes would not reach significance if one were to be applied. We did not apply a correction as our main aim was to test the association with the model parameters and the analysis of the behavioral measures was a supplementary analysis to check whether previous findings replicated. Given the higher risk of false positives, one could question whether the associations we observed between global efficiency and both mean RT and SD RT are “real” effects. We note that the associations we observed are consistent with the literature. Furthermore, in our random-subnetwork analysis we observed consistently negative correlations across subnetworks calculated for randomly selected brain regions. While these are not independent samples due to the correlation in white-matter characteristics across the brain, our results are not dependent on the selection of certain tracts.

In our study we investigated structural brain networks. A similar analysis can be done with functional brain networks derived either via functional MRI or via electrophysiological methods such as magnetoencephalography or electroencephalography. In principle, that should be done with data collected from recordings taken while participants are engaged in the task of interest. However, due to the correspondence between resting-state networks and task-related networks (Smith et al., 2009), valuable conclusions could be derived if resting-state functional connectivity were to be investigated.

## 5 Conclusions

The aim of our study was to leverage advances in the measurement and modeling of white-matter characteristics and use a model of the cognitive processes underlying speeded choice tasks to better understand the relationship between reaction time and structural connectivity in the brain. In agreement with previous studies, we find that the mean RT and RT variability are associated with network characteristics, which is consistent with proposals that efficient communication between brain regions supports the speed and consistency of behavioral responses. However, when we use a cognitive model to dissociate the process that underlies performance, the correlations disappear. These findings can be reconciled if we consider the psychometric properties and interpretation of the model parameters relative to the behavioral measures. While mean RT and SDRT may be theoretically less satisfying measures than those derived from the model, their reliability and generalizability across tasks give them predictive value.

## Supplementary Material

Supplementary Material

## Data Availability

The WAND data are freely available at https://doi.org/10.12751/g-node.5mv3bf. MRtrix and the Brain Connectivity Toolbox, which were used for the analysis of the MRI data, are freely available packages.
