# Supplementary material for: Composite reaction time and variability correlate with whole-brain white-matter characteristics
Source: Imaging Neurosci (Camb). 2026 Jul 17;4:IMAG.a.1303. doi: 10.1162/IMAG.a.1303 (PMC13382963; doi:10.1162/IMAG.a.1303)
Supplement: Supplementary Material [file IMAG.a.1303_supp.pdf]

Supplementary Material for:

Composite reaction time and variability correlate with whole-brain white matter characteristics

Eirini Messaritaki[1,*], Craig Hedge[1,2,*], Pedro Luque Laguna[1], Carolyn B McNabb[1], Derek K Jones[1], Petroc Sumner[1,]**

[1] Cardiff University Brain Research Imaging Centre, School of Psychology, Cardiff University, Cardiff, UK

[2]School of Psychology, Aston University, Birmingham, UK

[*]These two authors contributed equally to the work described in this manuscript.

[**] Corresponding author

S1. Variation of drift rate according to stimulus type

In our main analysis, we made the simplifying assumption that drift rate does not vary between stimulus type for the purposes of model fitting. We did this so that we could obtain a single estimate of "processing speed" per task so that each task was equally represented in the factor analysis. A reviewer requested that we test this assumption because inappropriate constraints imposed during the fitting process could lead to parameters being misestimated. We refit the two-choice reaction task allowing drift rate to vary for face and scene stimuli. Because we are primarily interested in individual differences, we were primarily concerned with the correlation between estimates obtained from the single drift-rate model and the two-drift rate model. These all exceeded .998 (see Table S1). Note that the table contains the correlation between drift rate from the single drift model with the *average* of the two drift rates from the two-drift model, though drift rates for faces and scenes within the two-drift model were also highly correlated (r=.981).

Table S1: Correlation between parameter estimates for the two-choice (face/scene discrimination) task from a model assuming a single drift rate and a model assuming separate drift rates for face and scene stimuli.

Parameter	r
Average drift rate	.999
Boundary separation	.998
Non-decision time	.999

For completeness, we also compared the model fits to evaluate whether allowing drift rates to vary improves the model fit. We see a slight improvement in the average BIC for the two-drift model (BIC=1475.96) compared to the single-drift model (BIC=1476.10), though this does not exceed typical rules of thumb for accepting the more complex model (i.e., a change in BIC of greater than 4). Therefore, our original assumption appears to be appropriate.

S2. Relationships between graph theoretical metrics

The correlations between the three graph theoretical metrics for the 4 types of networks (i.e., the combinations of whole-brain/task-specific and vNS/MWF) were high and statistically significant (see

Fig. S1).

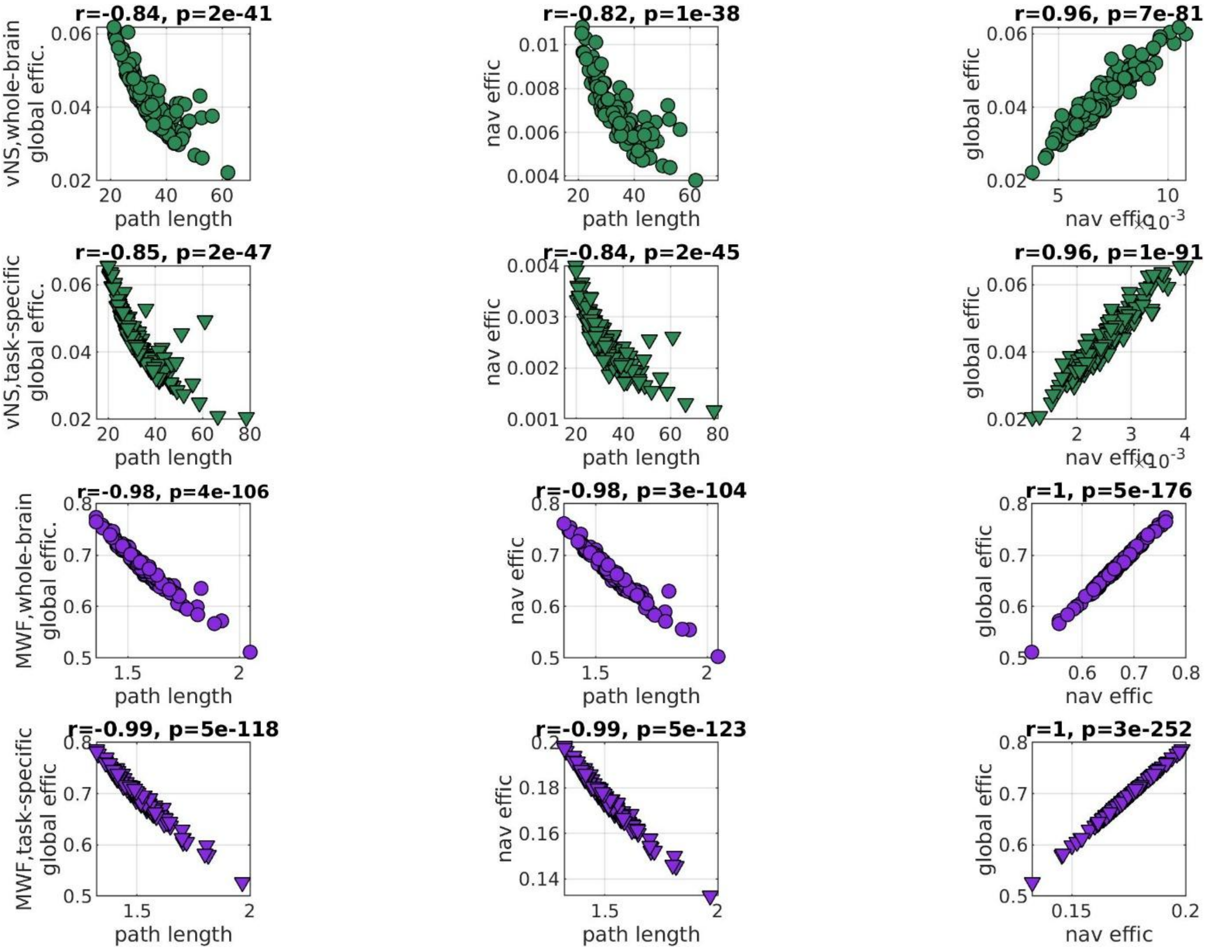

Figure S1: Correlations between the 3 graph theoretical metrics of interest for each of the 4 (sub)networks considered in this analysis. Abbreviations: nav effic = navigation efficiency, global effic = global efficiency, vNS = volume-normalized number of streamlines, MWF = myelin water fraction.

S3. Parameter recovery correlations

Parameter recovery simulations were conducted by simulating data for each task using the estimated parameter values from each participant. The number of simulated trials was equal to the number of trials administered in our tasks. We then fit the model to the simulated data and examined Pearson's correlations between estimated parameters and those used to simulate the data.

Table S2: Parameter recovery correlations for the three reaction time tasks.

	Task		
Parameter	Two-choice	Four-choice	Simon
Drift rate	0.72	0.69	0.96
Boundary	0.91	0.95	0.87
Non-decision	0.85	0.91	0.98

S4: Multiple linear regression results using individual task variables as predictors instead of composite scores

Note that in these analyses, the cognitive variables were the predictors rather than the outcome (as in our main analysis). This was necessary because multiple linear regression cannot accommodate multiple outcome variables.

Table S3: Standardized estimates from multiple linear regressions predicting global efficiency (vNS data) using cognitive variables as predictors.

		Whole-brain network		Task-specific network	
Predictors	Task	β	*p*	β	*p*
Drift rate	Two-choice	-0.03	.789	-0.02	.826
	Four-choice	0.00	.966	-0.01	.955
	Simon	0.10	.294	0.13	.187
Boundary	Two-choice	0.02	.811	0.03	.753
	Four-choice	**-0.29**	**.004**	**-0.29**	**.005**
	Simon	0.10	.290	0.07	.468
Non-Decision	Two-choice	0.01	.909	-0.02	.847
	Four-choice	-0.08	.388	-0.07	.459
	Simon	0.12	.187	0.09	.307
Mean RT	Two-choice	-0.08	.522	-0.09	.462
	Four-choice	-0.13	.221	-0.12	.268
	Simon	-0.04	.709	-0.01	.916
SDRT	Two-choice	-0.18	.120	-0.19	.093
	Four-choice	-0.12	.229	-0.11	.278
	Simon	0.03	.755	0.07	.502
Accuracy	Two-choice	**-0.38**	**.002**	**-0.35**	**.004**
	Four-choice	0.04	.739	0.00	.968
	Simon	**0.39**	**<.001**	**0.39**	**<.001**

Note. Significant associations (p<.05, uncorrected) are highlighted in bold.

S5: Multiple linear regression results using EZ-diffusion model parameters

Table S4: Standardized estimates from linear regression (for factor scores) and multiple linear regressions (for individual task variables) predicting global efficiency using EZ-diffusion model parameters as predictors.

		Whole-brain network		Task-specific network	
Predictors	Task	β	*p*	β	*p*
Drift rate	*Factor score*	*0.10*	*.216*	*0.07*	*.354*
	Two-choice	-0.16	.207	-0.13	.277
	Four-choice	0.03	.779	0.01	.902
	Simon	0.23	.051	0.19	.104
Boundary	*Factor score*	*-0.11*	*.152*	*-.098*	*.222*
	Two-choice	**-0.33**	**.002**	**-0.34**	**.002**
	Four-choice	0.04	.695	0.04	.680
	Simon	0.16	.079	**0.21**	**.028**
Non-Decision	*Factor score*	*-0.12*	*.150*	*-0.12*	*.124*
	Two-choice	-0.06	.618	-0.08	.491
	Four-choice	-0.13	.234	-0.11	.336
	Simon	-0.03	.802	-0.02	.866

Note. Separate regressions were conducted for each outcome. Significant associations (p<.05, uncorrected) are highlighted in bold.

S6. Including age as a moderator

Table S5: Standardized estimates from multiple linear regressions predicting each cognitive outcome by global efficiency, age, and the global efficiency x age interaction.

		Number of streamlines by volume				Myelin water fraction			
		Whole-brain network		Task-specific network		Whole-brain network		Task-specific network	
Outcome	Predictor	β	*p*	β	*p*	β	*p*	β	*p*
Drift Rate	Global Eff.	0.12	.123	0.15	.068	-0.01	.896	-0.03	.744
	Age	**0.34**	**<.001**	**0.36**	**<.001**	**0.33**	**<.001**	**0.34**	**<.001**
	Interaction	-0.02	.821	0.02	.848	**-0.24**	**.023**	**-0.28**	**.010**
Boundary separation	Global Eff.	0.03	.706	0.05	.454	0.03	.936	0.01	.844
	Age	**0.38**	**<.001**	**0.40**	**<.001**	**0.41**	**<.001**	**0.41**	**<.001**
	Interaction	0.13	.202	0.13	.244	-0.11	.298	-0.15	.213
Non-decision time	Global Eff.	-0.02	.812	-0.06	.488	-0.07	.508	-0.12	.243
	Age	-0.13	.124	-0.13	.119	-0.11	.236	-0.10	.263
	Interaction	0.02	.811	-0.05	.664	0.13	.274	0.09	.457
Mean reaction time	Global Eff.	-0.13	.095	-0.11	.153	0.07	.399	0.03	.729
	Age	**0.31**	**<.001**	**0.31**	**<.001**	**0.30**	**<.001**	**0.31**	**<.001**
	Interaction	-0.05	.541	-0.03	.681	0.11	.282	-0.01	.918
SD of Reaction time	Global Eff.	**-0.22**	**.007**	**-0.20**	**.017**	0.14	.127	0.13	.162
	Age	0.03	.750	0.02	.851	0.02	.797	0.03	.745
	Interaction	-0.13	.139	-0.13	.155	0.13	.249	0.05	.652
Accuracy	Global Eff.	0.04	.620	0.05	.513	0.01	.857	-0.02	.801
	Age	**0.37**	**<.001**	**0.38**	**<.001**	**0.35**	**<.001**	**0.35**	**<.001**
	Interaction	0.10	.286	0.11	.229	-0.07	.489	-0.14	.220

S7. Mediation analysis for reaction time variability

We conducted a mediation analysis to test whether global efficiency mediated the association between age and SDRT, complementing our analysis of mean RT in the main text (see Figure 6). The indirect effect was not statistically significant in either task-specific network data or the whole-brain data. Notably, the paths between age and global efficiency and between global efficiency and SDRT were significant in both analyses, but the product of these (the indirect effect) was not sufficiently large to reach significance.

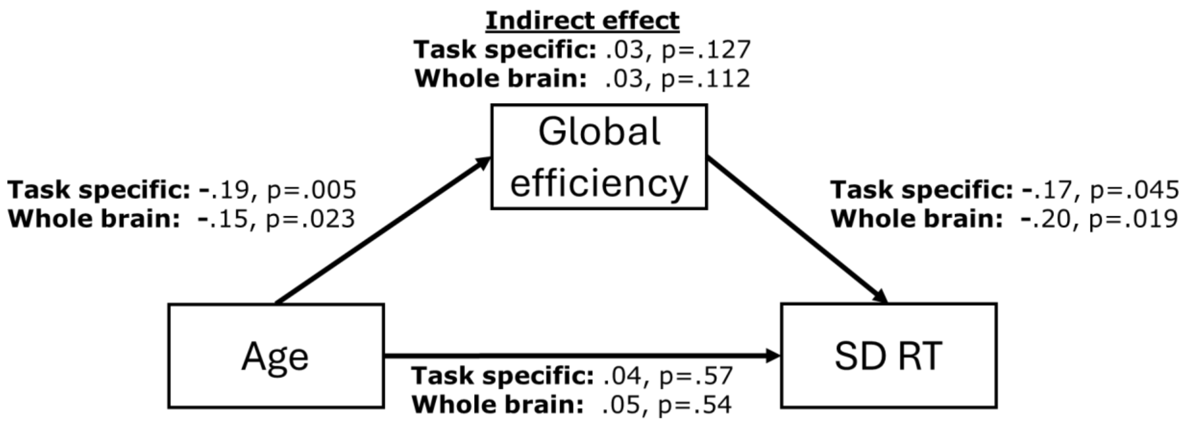

Figure S2: Standardized regression coefficients for the relationship between age and standard deviation of reaction time as mediated by global efficiency.
